# Extra Supplement—The Nursing Mirror

**Published:** 1890-04-26

**Authors:** 


Being the Extra Nursing Supplement of "The Hospital" Newspaper.

All Contributions for this Supplement should be addressed to the Editor, The Hospital, 140, Strand, London, W.C., and should have the word "Nursing" plainly written in left-hand top corner of the envelope.

### En Passant.

**THE BICESTER HOME.**—During the past year there have been 17 patients treated at the Home, and the two nurses have visited 118 poor people in the town; 16 private cases have also been nursed. The receipts were £196, and there is a balance in hand of £58. We congratulate the committee on the work done, and on such a pleasant balance-sheet.

**THE QUEEN'S NURSES IN WALES.**—The small pamphlet issued by the Welsh branch of the Jubilee Institute is a model of clearness and conciseness. It begins with a vigorous description of what district nursing is, explains the necessity for such nursing, tells how the work can be carried out, and concludes with a terse demand for funds. We hope the funds will be forthcoming to maintain the work which has been begun, at 11, The Parade, Cardiff, where a superintendent (Miss Heath) and two nurses are now at work.

**SHORT ITEMS.**—We regret that, owing to a rush of Easter work, the name of Miss Yate lately appeared in these pages as Miss Yale, and the name of Miss McManus as Miss McMasters.—At the end of the inquiry as to the death of the patient Seagrave at Drogheda Infirmary, the doctors, nurses, and officers of the institution were censured. —We are glad to hear from Sister Frost that the Brassey Home is in no way sectarian, and that attendance at prayers is not compulsory. The fullest possible freedom in all matters is aimed at.—Mr. Henry Tate, of Liverpool and London, has given £10,000 towards furthering district nursing in these two towns.

**NEWS FROM KALIHI.**—The following is an extract from a letter lately received from Sister Rose Gertrude: "This is not the hospital I was originally intended for, but is newly-built, and we have the German specialists here. There are already forty patients, and they needed an English sister very much, as the natives will not pursue their treatment if left to themselves. This is a very pretty spot, built on the coral; the patients are in little detached wooden cottages, all around a green lawn, with shady trees. In time we are to have many pretty flowers and creepers. I have some little children here, so that my work is teaching as well as nursing, and amusing them all as well. There is one drawback: we are a long way from the church, and I hope in time to get a little chapel where I may assemble them for prayers, reading, and singing. I have a German patient, who feels his misfortune very much . . ."

**THE RURAL NURSING ASSOCIATION.**—The work of this Association has recently received a most gratifying proof of interest and sympathy from a kindred association—the Ladies' Sanitary Association, London—in the shape of a donation of fifty guineas. The Sanitary Association has given this generous evidence of sympathy in the belief that much of the work they desire to see done in the country districts in the efficient nursing of the sick poor and in sanitary instruction will be carried out by the Rural Nursing Association. This association has been lately strengthened by the interest of the following ladies, who have joined it as vice-presidents: The Countess Bathurst, the Countess Beauchamp, the Countess of St. Germans, Lady George Hamilton, the Viscountess Lifford, Viscountess Ebrington, Lady Louisa Fortescue, Lady Susan Fortescue, the Lady Fitz-Hardinge, the Lady Sudeley, the Lady Montagu, Mrs. Francis Jeune, and Mrs. W. H. Smith.

**ST. JOHN'S HOUSE.**—How many of us know this house in Norfolk Street, and how many more of us know the quaint (not to say ugly) brown uniform of the nurses, which we have grown to love for the sake of sweet associations. St. John's House has fallen on comparatively evil days, but through many trials its steady growth has gone on unceasingly, and if Charing Cross has been lately lost, the Metropolitan Hospital has been lately won. The community has also a maternity home, a district nursing branch, and a supply of private nurses. The committee are just now appealing for increased support, chiefly to aid the maternity home at Battersea, and to increase the number of district nurses. We hope their appeal will be successful, for a home started in 1847 deserves sympathy.

**BEEF TEA.**—In the last number of the *Lancet* Dr. Arthur P. Luff gave the analyses of beef-tea and some other preparation of beef, in which he proved that the preparations are equally strengthening to the rumpsteak beef-tea. This is a point of the greatest interest to nurses, and we gladly quote the following paragraph from the article in question: "These analyses show that only a very small quantity of proteid or albuminous matter is present in all the beef-teas examined, and also that the beef-teas made from bouillon Fleet and from Kemmerich's peptone of beef are throughout stronger in composition than the best beef-tea made by the ordinary process from rumpsteak, while the beef-tea made with one-quarter of a teaspoonful of Kemmerich's extract of beef to a cupful of water is slightly weaker than ordinary beef-tea. We have, therefore, in beef-tea prepared either with Bouillon Fleet or with peptone of beef a preparation which is stronger as regards every import constituent than ordinary beef-tea, while it can be prepared at one-quarter to one-third the cost, together with a saving of the labour involved in making ordinary beef-tea. The comparative cheapness of these preparations is easily understood when we consider the great difference in the price of beef in this country and in South America."

**BRITISH NURSES.**—Apparently the entries for the central register are not so numerous as were expected, for we see from the report of the British Nurses' Association that once more the time of grace is extended for the admission of uncertificated nurses. From the figures given it appears that only 862 nurses out of the more than 3,000 members which the British Nurses' Association claims have applied for registration. In other words, only about 25 per cent. of the members of the Association have, as yet, gone upon the register; does this mean that the others have ceased to belong to the Association, and that the statements that numerous withdrawals have and are taking place are true? Rather late in the day the notion of a Royal Charter is apparently abandoned, and the Association is going to follow the example of the Midwives' Institute and apply for incorporation only under the Companies Acts. The Association have decided to abandon insistance on a period of three years' training in favour of "one year in a recognized general (? cottage) hospital with, at least, 40 beds," and two years subsequently in "hospital work," whatever that may mean. Even these modified requirements may be waived so far as the "twelve months" in a "recognized hospital" is concerned, at the discretion of the Registration Board. These regulations will not take effect before 1st July next, and meanwhile all and sundry may apply for registration. Yet—or is it because of this lowering of the standard?—the 862 are described as "the *elite* of the nursing profession!"

### Nursing Lectures.

By Percy Lewis, M.D.

### Lecture IX.—THE KIDNEYS.

The kidneys, two in number, are placed deeply in the abdomen on the posterior wall, so deeply, in fact, that from a nursing point of view they may be considered as being placed in the back, one on each side of the spinal column, and extending from just above the bony pelvis to behind the eleventh and twelfth ribs. They secrete those substances which are of no further use to the body, substances which, if retained, would poison the individual. If the kidneys stop secreting, as in some diseases they do, the patient will become comatose, and, unless relieved, will die.

In structure the kidneys consist of numberless little microscopic tubes, covered on the outside with capillary blood vessels, on the inside with cells, having at the extreme end a filtering apparatus, by which the water is strained off from the blood. As the water passes down the little tubes it dissolves and carries with it the solid matter which has been secreted.

The functions of the skin and kidneys are very much like each other, both secreting water and waste matter. If the kidneys are diseased we endeavour to make the skin do more work, thus giving the kidneys less, and, therefore, putting them in a more favourable condition for recovery.

The kidneys are liable to many diseases, some of which we will briefly notice. The solid matter may separate out in the kidneys and so increase in amount as to form a stone. This may either remain there, giving rise to inflammation or abscess, or it may pass on, giving rise to that intense agony called renal colic. Inflammation of the kidney is spoken of as Bright's disease, which may be either acute or chronic. A floating kidney is one which has become detached from its normal position, and is more or less loose in the abdomen. The special symptoms of acute Bright's disease are rise of temperature, aching in the loins, hot dry skin, rigors, vomiting, and a reduced quantity of urine which contains blood. In chronic Bright's disease, the digestive organs are out of order; from affections of the blood vessels the patient becomes liable to hœmorrhages, as nose bleeding and hamatemesis, and œdema, or dropsy of various organs; or even of the whole body may set in with rapidity.

Now, there is one great danger to which all cases of Bright's disease are liable, viz., uræmia. Uræmia is the state produced by retention of the constituents of the urine in the blood. The object of treatment in these cases is to make the skin and intestines do the work which the kidneys are unequal to. As long as we are successful in this uræmia is avoided. If these secretions are stopped by cold or by constipation, the constituents will accumulate in the blood, and uræmia will be produced more or less quickly. As a rule it does not come on suddenly, but is preceded by certain premonitory symptoms, which a nurse should know, be always on the look-out for, and report at once. Treatment applied early can generally ward off the attack, but the later applied the less hopeful it will be. Some of these symptoms are so slight that they may be missed altogether by a doctor at a short visit, and unless you had been warned of their importance you might neglect to report them. The slighter ones are headache, noises in the ears, slight impairment of vision, drowsiness, vomiting, and slight—often very slight—twitching of muscles of face and hands. These, if not treated, will be followed by delirium, convulsions, dyspnœa, insensibility, and death. The insensibility may be continuous, and accompanied by fits, or interrupted by periods of sensibility free from symptoms. I have notes of a case of this sort, who had forty-four fits in one day, and yet recovered under treatment.

As far as general nursing is concerned warmth is most essential. A renal patient should have flannel night shirts, and should sleep between blankets. As these night shirts soon become saturated with perspiration they should be often changed, and the skin should be daily washed with warm water, a small portion only being uncovered at a time. The vitality of the skin becomes lowered, partly by the excessive work which it has to do and partly by its chronically œdematous condition. On account of its lowered vitality it becomes liable to bedsores, which heal with difficulty, and slight scratches or cuts are very liable to be attacked with erysipelas.

Cough in renal cases probably points to commencing œdema of the lungs; in fact, any of the symptoms of chest disease become doubly important, and are to be specially looked for and reported. A nursing point frequently forgotten is the position of the patient. As dropsy tends to affect the most dependent parts, it follows that if a patient is up the legs will be most œdematous; but if he is in bed the brain, lungs, and heart will stand an equal chance. And the danger from this cause will more than counterbalance what is gained by rest and warmth. The danger is to be averted by making the patient sit up as much as possible in bed, and trying to get him to sleep in this position; at any rate, for every hour he lies down he should spend an equal time sitting up.

The diet is a very important part of treatment. As a rule, butcher's meat in any form is to be avoided, and in acute Bright's disease milk and sodawater may be the only diet allowed. Patients may be kept on nothing but milk, which in many cases holds out the only hope of cure. In more chronic cases, and especially in private cases where people refuse to take milk diet, there are several other articles of food which may be allowed. Fat, for instance, in any form as bacon, cream, butter, also liver, vegetable stews, biscuits, salads, fruits, tea, coffee, porridge, and milk puddings without eggs. The latter may be flavoured with ginger, cloves, or cinnamon. From this limited dietary it will be necessary to make as many variations in the meals as possible. The more variety you can think out the longer will a patient be content to continue it, and the more chance, therefore, of his ultimate cure.

The medicinal treatment of Bright's disease consists in stimulating the other organs to do more work, and so make up for that neglected by the damaged kidneys. Purges are given with this object, drugs to produce sweating, diaphoretics, as they are called; and, lastly, the kidneys themselves are stimulated by drugs called diuretics. When there is much œdema the superfluous water is got rid of by puncturing the legs with a lancet, or by inserting little tubes —"Southey's tubes"—through which the water can drain. These measures are supplemented by hot packs, hot-air baths, and other means to be described later on.

---

### Appointments.

[It is requested that successful candidates will send a copy of their applications and testimonials, with date of election, to The Editor, The Lodge, Porchester Square, W.]

Devonshire Square.—Miss Worsnup, who trained at Westminster, has been appointed lady superintendent of this institution in place of Mrs. Keeley, resigned.

Maryborough Hospital.—Miss Martelli, who has been head nurse in the Sick Children's Hospital, Melbourne, resigned her post in January. She received early in March the appointment of matron to the hospital at Maryborough, Australia. Miss Martelli is a great favourite, and holds most excellent testimonials, especially from the Alfred Hospital, where she was trained. We send our best wishes and most hearty congratulations to this friend across the seas.

Gorleston Cottage Hospital.—Miss C. B. Wilkie of the Cottage Hospital, St. Helen's, Lancashire, has been appointed matron at Gorleston.

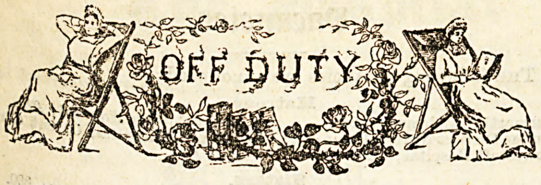

### The Passing of the Shadow.

II.

In an upper chamber of the same chateau sat Annette, Sophie's youngest sister. The room was large, and led from one side by a short staircase into the garden, from the other into the chateau itself. The walls of the room were slightly tinged with green, there were no hangings over the windows or the doors, and the furniture, of light green and gold, was in the smooth self-contained style of Louis XVI. A tiny lamp burnt on a beautiful inlaid table; but the room, though furnished with exquisite taste, would have been somewhat prim and bare, had it not been for the presence of the young girl sitting by the window and watching the sun setting. The dying light brought out the rich tones of her face, upturned to catch its rays. She was very beautiful—of that soft indescribable beauty, which, like that of the sublimest poems, is lost in analysis, and which at this moment was intensified by her absorption in the scene around her. It was certainly worthy her attention.

The fields and lakes, on a lower level than the chateau, lay with bosom open to the sky. No clouds broke its vast expanse; only the misty golden light faded imperceptibly into the blue, high up; the lakes placidly absorbed the light, which was fiercely white, and shadows rested upon the hills. There was something terrible in the calm intensity of the sunset as it burned itself away; something awestruck in the passive landscape, as it strove to hold the golden mist; the air was big with absolute silence. Half trembling at the great beauty, half with a rapturous and dreamy joy, the girl drank in the golden light. But now the film of death was gathering over the day. Paler and paler grew the lakes, darker and darker the hills, and the low rustle of a wind in the trees, very close, told that night was near. The misty light rose in curves like incense into the blue; the stillness became softer and less strained, and the girl, exhausted with the intensity of her feeling, saw the last light on the lakes die out, and felt that the soothing peace of twilight was covering the land.

The lamp, after many ineffectual efforts to proclaim itself, is now smiling mildly at its own success.

O God! what is that shadow it casts upon the wall, as the girl half turns her head to avoid the cold night breeze? It has slid in from the door on the side of the chateau, and the great black thing moves slimily, cautiously, along the wall. Why is it that all her limbs are paralysed, and that she feels faint with a feeling of sick horror? Why can she not speak in turn, but must sit there with her eyes nailed on the flat moving mass? She cannot distinguish the form, but the slow creeping movement creates in her an intense loathing. It is passing to the other door, it will soon be gone.—O turn before it is too late—do not be haunted by shadows all your life—prove that it is a dream, or more horrible still, that it is no dream! With a violent effort, Annette half rose from her chair, strove to speak, but the words would not come, and a mist swam before her eyes.

When next she looked round, the shadow was gone, and her feelings and powers, unnaturally petrified, returned to full activity. She ran almost frantically to the staircase, down which the horrible thing had disappeared, descended it, and went into the garden; she saw nothing there but the dim forms of the shrubs in the faint blue of the moon. She went up to her room again to call some of the people of the chateau. There she almost fell into the arms of her sister Celimène—into whose face she had but to glance to know that something terrible had happened. "Our brother, he is dead," sobbed Celimène, "he is murdered!"

"And I," said Annette, as pale as death, "have seen the murderer."

*(To be continued.)*

### Everybody's Opinion.

*[Correspondence on all subjects is invited, but we cannot in any way be responsible for the opinions expressed by our correspondents. No communications can be entertained if the name and address of the correspondent is not given, or unless one side of the paper only be written on.]*

### THE JUNIUS S. MORGAN MEMORIAL.

Nurse Chapman writes: It is with sad regret I read in The Hospital the death of Mr. Morgan. My sister nurses and myself at the Lincoln Institution have so wished we could have shown our gratitude to our great benefactors and friends of nurses, and now one has gone to receive his reward. I shall watch with interest what is proposed to be done to honour such a good man. I feel sure many others will be happy as I myself shall be to do what I can.

Nurse D. writes: It would give me, and I am sure many other sick nurses, great pleasure to testify our respect and gratitude to the late Junius Morgan for his share in promoting the Pension Fund. We have, indeed, reason for thankfulness in a scheme that enables us to provide for sickness and old age without banishment to almshouses, or the humiliation of soliciting votes and private interest. Such tribute to be grateful must be speedy. Should not the first thousand show the way? We cannot, I fear, raise enough to endow a cot, that best form of memorial, but a thousand half-crowns, or even a thousand shillings would pay for a stained glass window or ornament of that sort to brighten one of the gloomy chapels of the London hospitals, to the glory of God and in pious memory. As you will, no doubt, have many letters on this subject, I need say no more.

Six Nurses write: The nurses of St. John the Divine beg to express their deep sympathy with the family and council at the loss of Mr. Junius S. Morgan, and would be most happy to contribute towards a fitting memorial to one who has done so much for nurses.

Four Private Nurses write: We feel sure that the nurses will gladly come forward to show their gratitude to their kind and generous benefactor, the late Mr. Junius S. Morgan, who, in conjunction with other of the nurses' best friends, has given them the surety that with a little prudence on their part the days of sickness and old age will be lightened of half their burden. Many of us read with sorrowful hearts of the fatal accident that deprived us of our good and noble-hearted friend, and though we knew him only by his deeds, we feel that we are expressing the thoughts of many nurses when we say how deeply we sympathise with those who personally knew and loved him, and in whose circle there is now missing a dear and honoured face.

A Scotch Nurse writes: I am truly grieved to hear of the death of one of our best friends. Could not the first thousand take up his work and help their poorer sisters?

[We have received other letters and other suggestions, which are too numerous to be produced. If the First Thousand will collect what they can by June, this will be the best memorial. Those who cannot collect £5 alone can join, two or three together, to form one purse between them.]

### TWO SUGGESTIONS.

"S." writes: I would suggest that nurses belonging to no institution, like myself, should wear pretty washing frocks, made in a simple style, to suit the wearer, with a becoming cap and apron. Thus we may make a suitable appearance to come before our beloved Princess. From a sanitary point, I think nurses should cultivate a taste for wearing washing materials. I have read with sorrow and deep interest your account of Mr. Junius Morgan's death. Truly we have lost a kind friend and benefactor. I am sure, individually and as a body, the nurses will all wish to offer their sympathies to his family, in appreciation of his great kindness to us.

### ASYLUM NURSES.

P. B. writes: My attention was drawn to an answer to one of your correspondents in last month's issue of The Hospital, in which it was stated that asylum nurses were of a lower status than were hospital nurses, and that asylum nurses were taken entirely from the "servant" class of women. This is quite an erroneous idea, and I will feel obliged if you would kindly allow me space in your paper to correct the statement. My object in writing is to try to remove the false impression which such a statement would naturally leave on the minds of persons who have no experience or knowledge of the facts of the case—an impression which would, I fear, have the effect of preventing many from becoming nurses, who would probably prove valuable assistants. I have no intention whatever of speaking disparagingly of domestic servants; on the contrary, I believe them to be a respectable and praiseworthy class, and I would as willingly associate with a respectable servant as I would with the daughter of a professional gentleman. Still I know there are many who, from a feeling of false pride, refuse to "mix" with servants, and it is for this reason that I would wish to give my experience of the kind of girl engaged as asylum nurse. I am one of a staff of forty-one nurses, out of which number not more than six have been engaged as servants. The majority of the nurses here are farmers' daughters—girls who have never been from home before. Amongst us, too, we have a clergyman's daughter, and we have had as nurses the daughters of more than one medical gentleman. For myself, I may say that before filling my present office I was for more than six years teacher in a large public school, and I find that most of my companions here are as respectable and well educated as were my companions in school.

### MALE NURSES.

A Male Nurse writes: I read in a recent number of The Hospital that someone protested strongly against the title of nurse being used by us, and that we ought to be designated as sick attendants, orderlies, etc., at the same time scorning the idea that nursing could be done by us. Now we are a small body (*i.e.* certified nurses) and no attempt is made by us to interfere with the ministrations of women, who are often very glad of our assistance when patients are delirious, etc., and I cannot close when I see masseuses advertising for lady or gentlemen patients without quoting what an author of a book on massage says that: "I am sorry to say that facts are coming to my knowledge which are detrimental in the highest degree to the best interests of massage. I refer specially to the growing practice of masseuses operating upon male patients. This is very wrong in every way, and should be discouraged in the most determined and positive manner by all members of the profession. It is immoral, disgusting, degrading, and utterly unjustifiable."—T. S. Dowse, M.D. At the same time I must admit that some of us—more often men who are really lunatic attendants and nothing more—are quite unfit for some cases which require very careful nursing, management, and sympathy. I should be glad to hear the views of other male nurses on this subject.

### A CORRECTION.

Penzance writes: I see in a late Hospital you have an inaccurate report of the work of the nurses of this town. Our report is, a room is opened two afternoons a week (not at the nurses' home), at which the nurse attends where those suffering with bad legs in the town and neighbourhood can go to have them dressed. The charge of one penny each attendance is made, which the patient gladly pays, to go towards the fund for providing strapping, ointment, &c. This enables those outside the town, who otherwise are not entitled to the nurses' services to have the benefit of a good dressing and bandaging twice a-week. It is also a great saving of the nurses' time, which is often needed for more serious nursing. Our plan has answered admirably, and is worked by the sanction of the doctor.

### Notes and Queries.

### Queries.

(4) *Nursing at Singapore.*—Can anyone give information as to whom to apply for particulars?—*Dodo.*

(5) *Home Wanted.*—Can anyone tell me of a Home within twenty miles of Bristol, near sea preferred, for a young man paralyzed. Can pay about 10s. or 12s. per week. Would wish for companionship, and to be allowed to amuse himself by various kinds of work. Answer to Superintendent, Nurses' Institute, Weston-Super-Mare.

### Answers.

*Sergeant Fagg.*—We never prescribe. Consult a medical man.

*Ataton.*—Will appear shortly.

*Sister-in-Charge.*—The committee are not bound to give their reasons for discharging a matron, though it is usual and more just to do so.

*Mrs. F. L. E.*—Your suggestion is good, but it will be well to carry out the larger scheme first. We hope for your help in the scheme, and then we will work for yours afterwards.

### Vacancies.

The following vacancies are announced:

### Matrons.

Carmarthen Infirmary; £40.

Hulme District Nurses' Home; £40.

Lowestoft Hospital; £50.

Tenbury Cottage Hospital; £30.

St. Peter's Hospital, Covent Garden; £60.

### Sisters.

Evelina Hospital.
Newcastle Royal Infirmary; £30.

### Nurses.

Aimee Nursing Homes.

Arbroath Poorhouse; £25.

Beckett Hospital; £24.

Blackheath Nursing Institute.

Bolton Infirmary; £25.

Boro' Hospital, Birkenhead; £25.

Brixton Institute.

Bromley and Beckenham Hospital; £25.

Burnley Victoria Hospital, £26.

Chelsea Infirmary; £18.

Cheltenham Hospital.

Clayton Hospital, Wakefield; £25.

Cottage Hospital, St. Helen's, Lancashire.

County Hospital, Dorchester (night); £22.

Croydon Hospital; £25.

Fever Hospital, Carlisle; £20.

Fever Hospital, Darlington; £26.

General Nursing Institute.

Glamorgan and Monmouthshire Infirmary, Cardiff; £28.

Glasgow Sick Poor and Private Nursing Association.

Greenwich Union; £20.

King's Cross Hospital, Dundee; £27.

Longton Cottage Hospital (night); £20.

Lynn Hospital; £20.

Mr. Wilson's Institution.

Newcastle Infirmary; £20.

Newport Institute; £20.

North-West London Hospital; £24.

Oldham Infirmary; £28.

Ophthalmic School, Hanwell; £26.

Rochdale Infirmary; £22.

Royal Portsmouth Hospital; £25.

Sheffield Union; £20.

Stratford-on-Avon Hospital; £20.

St. Saviour's Infirmary; £20.

Swansea Institute; £25.

The Hospital, Redhill, Surrey; £20.

Victoria Park Hospital; £22.

Wakefield Hospital; £25.

Westhampnett Union Infirmary; £22.

Whitechapel Infirmary; £20.

Workhouse Infirmary Nursing Association.

Worthing Infirmary; £24.

### District Nurses.

Ashton-under-Lyne Association.
Bolton, Lancashire; £30.
Bristol Nurses' Society.
Cambridge Home.
Chailey, Sussex; £30.
East London Nursing Society.
Exmouth Maud Hospital.
Gloucester Society; £25.
Jersey Institute; £25.
Queen Victoria Nurses' Institute, Edinburgh; £30.

### Midwives.

Barrow-upon-Soar.
Belfast Lying-in Hospital.

### Probationers.

Cottage Hospital, Wallasey, Cheshire.
Grimsby Hospital.
Highgate Hospital for Children.
H.M. Hospital for Waif Children, E.
Leeds Nurses' Institution; £14.
Liverpool Hospital for Women.
North-West London Hospital; £8.
Poplar Hospital for Accidents.
District nurses
Sheffield Union; £10.
Sydenham Children's Hospital.
Workhouse Infirmary Nursing Association.

### Amusements and Relaxation.

### N.B.—New Word Competition Commenced April 5th, 1890, ends June 28th, 1890.

Three prizes of 15s., 10s., 5s., will be given for the largest number of words derived from the words set for dissection.

Proper names, abbreviations, foreign words, words of less than four letters, and repetitions are barred; plurals, and past and present participles of verbs, are allowed. Nuttall's *Standard* dictionary only to be used.

N.B.—Word dissections must be sent in WEEKLY to 140, Strand, W.C., arranged alphabetically, with correct total affixed.

The word for dissection for this, the FOURTH week of the quarter, being

"PORTLAND."

| Names. | Ap. 17th. | Totals. |
|---|---|---|
| Adeline | 10 | 41 |
| Patience | 10 | 50 |
| Lightowlers | 10 | 48 |
| Canary | 7 | 40 |
| M. E. S. | — | 37 |
| Ambition | — | 36 |
| M. W. | 10 | 50 |
| Esperance | 10 | 50 |
| Judy | — | 37 |
| Reldas | 10 | 49 |
| Merenda | — | 29 |
| Lucy Locket | 8 | 40 |
| Camellia | — | 30 |
| Qu'appelle | 10 | 50 |
| Tinie | 10 | 50 |
| Nurse Mildred | — | 33 |
| Coralie | 9 | 48 |

| Names. | Ap. 17th. | Totals. |
|---|---|---|
| Gladys | 10 | 48 |
| Agamemnon | 10 | 50 |
| G. E. Y. C. | 10 | 34 |
| S. E. A. | — | 24 |
| Jenny Wren | 10 | 50 |
| Multum in parvo | — | 32 |
| Ecila | — | 39 |
| Weta | — | 26 |
| Henri | 9 | 44 |
| Holland | 10 | 50 |
| T. J. | 7 | 33 |
| Nurse Isabel | — | 17 |
| Nurse Faith | 9 | 9 |
| E. S. Z. | 10 | 10 |
| G. P. | 10 | 10 |
| Stumps | 10 | 33 |
| Bolton | 8 | 8 |

### Notice to Correspondents.

Stumps.—Total for first week, 23.

N.B.—Each paper must be signed by the author with his or her real name and address. A *nom de plume* may be added if the writer does not desire to be referred to by us by his real name. In the case of all prize-winners, however, the real name and address will be published.

Competitors can enter for *all* quarterly competitions, but no competitor may take more than *one first prize* during the year.

